# Supplementary material for: VPS39-deficiency observed in type 2 diabetes impairs muscle stem cell differentiation via altered autophagy and epigenetics
Source: Nat Commun. 2021 Apr 23;12:2431. doi: 10.1038/s41467-021-22068-5 (PMC8065135; doi:10.1038/s41467-021-22068-5)
Supplement: Supplementary file 1 — Supplementary Information [file 41467_2021_22068_MOESM1_ESM.pdf]

SUPPLEMENTARY INFORMATION to *VPS39*-deficiency observed in type 2 diabetes impairs muscle stem cell differentiation via altered autophagy and epigenetics

SUPPLEMENTARY METHODS

DNA methylation arrays

DNA methylation was analyzed in myoblasts and myotubes from 14 individuals with type 2 diabetes (T2D) and 14 control individuals using Infinium HumanMethylation450 BeadChip (Illumina). This array contains more than 450,000 CpG sites covering 99% of RefSeq genes [1]. Genomic DNA was bisulfite-converted using EZ DNA methylation kit (Zymo Research, Orange, CA, USA), and DNA methylation analyzed according to the Infinium HD assay methylation protocol guide (Illumina #15019519). Myoblasts and myotubes from one subject were always analyzed on the same chip, and the samples from controls and individuals with T2D were evenly distributed on the chips to reduce batch effects. The raw methylation score for each probe represented as methylation β-values was calculated using GenomeStudio Methylation module software. All included samples showed a high quality bisulfite conversion efficiency (signal intensity > 4000), and also passed all GenomeStudio quality control steps based on built in control probes for staining, hybridization, extension, and specificity. Before further analysis, the DNA methylation data was exported from GenomeStudio and subsequently analyzed using Bioconductor and the lumi package. β-values were converted to M-values. Next, data were background corrected by subtracting the median M-value of the 600 built-in negative controls, and was further normalized using quantile normalization. Data were further processed [2] using BMIQ [3] to correct for probe type, and COMBAT [4] for batch correction. COMBAT was not used for comparison of DNA methylation between myoblasts and myotubes from the same individuals since these samples were placed on the same chip. As a result, 5365 probes with a mean detection *p*-value > 0.01, 65 rs-probes, 86 Y-chromosome probes, 2835 ch-probes targeting non-CpG sites, 13,731 probes based on at least 49 cross reactivity [5], and 5020 probes with a MAF > 0.1 [6] were excluded.

DNA methylation was analyzed in VPS39-silenced myoblasts and negative control (*n* = 6 independent experiments), three days after the transfection with siRNA and the start of differentiation, using Infinium MethylationEPIC BeadChip kit (Illumina) that covers approximately 850,000 CpG sites. Paired samples (VPS39 knockdown and control from the same experiment) were analyzed on the same chip. DNA was amplified, fragmented, and hybridized to the BeadChips according to the Infinium HD assay methylation protocol, followed by imaging of the BeadChips with Illumina iScan after single base extension and staining. Raw fluorescence intensities were extracted to obtain raw methylation score for each DNA methylation site. All samples passed the quality control based on built-in control probes for staining, hybridization, extension and specificity. Bisulfite conversion efficiency was of high quality (signal intensity > 4000). DNA methylation data were exported from GenomeStudio, and converted to M-values. Quantile normalization, background-correction, and BMIQ [3,7] were performed to analyze the data using Bioconductor [8]. Targets for probes with a mean detection *p*-value > 0.01, rs-probes, Y-chromosome probes, probes based on at least 49 cross reactivity [9], and probes with a MAF > 0.1 were removed from the initial analysis. Data for 817,702 CpG sites remained, 95,167 of which mapped to genes with differential expression.

All methylation data are presented as β-values ranging from 0 to 1 (0-100% methylation).

qPCR

Extracted RNA was converted to cDNA by using QuantiTect reverse transcriptase kit (Qiagen #205311). qPCR was performed using pre-designed TaqMan gene expression assays (Applied Biosystems, ThermoFisher Scientific) or SYBRgreen primers (DNA Technology A/S, Risskov, Denmark), and detected using the ViiA 7 real-time PCR system or QuantStudio 7 Flex Real-Time PCR system (both from Applied Biosystems). Expression levels were normalized to those of the housekeeping genes *UNC50* and *PPIA* (human), or *Ppia* and *Gusb* (mouse). Samples were run in triplicate, and the expression quantified using the standard curve method (human), or $2^{-\Delta\Delta Ct}$ method (mouse) using the geometric mean of the housekeeping genes for normalization. All primers used are listed in Supplementary Table 2.

SUPPLEMENTARY FIGURES AND FIGURE LEGENDS

SUPPLEMENTARY FIGURE 1

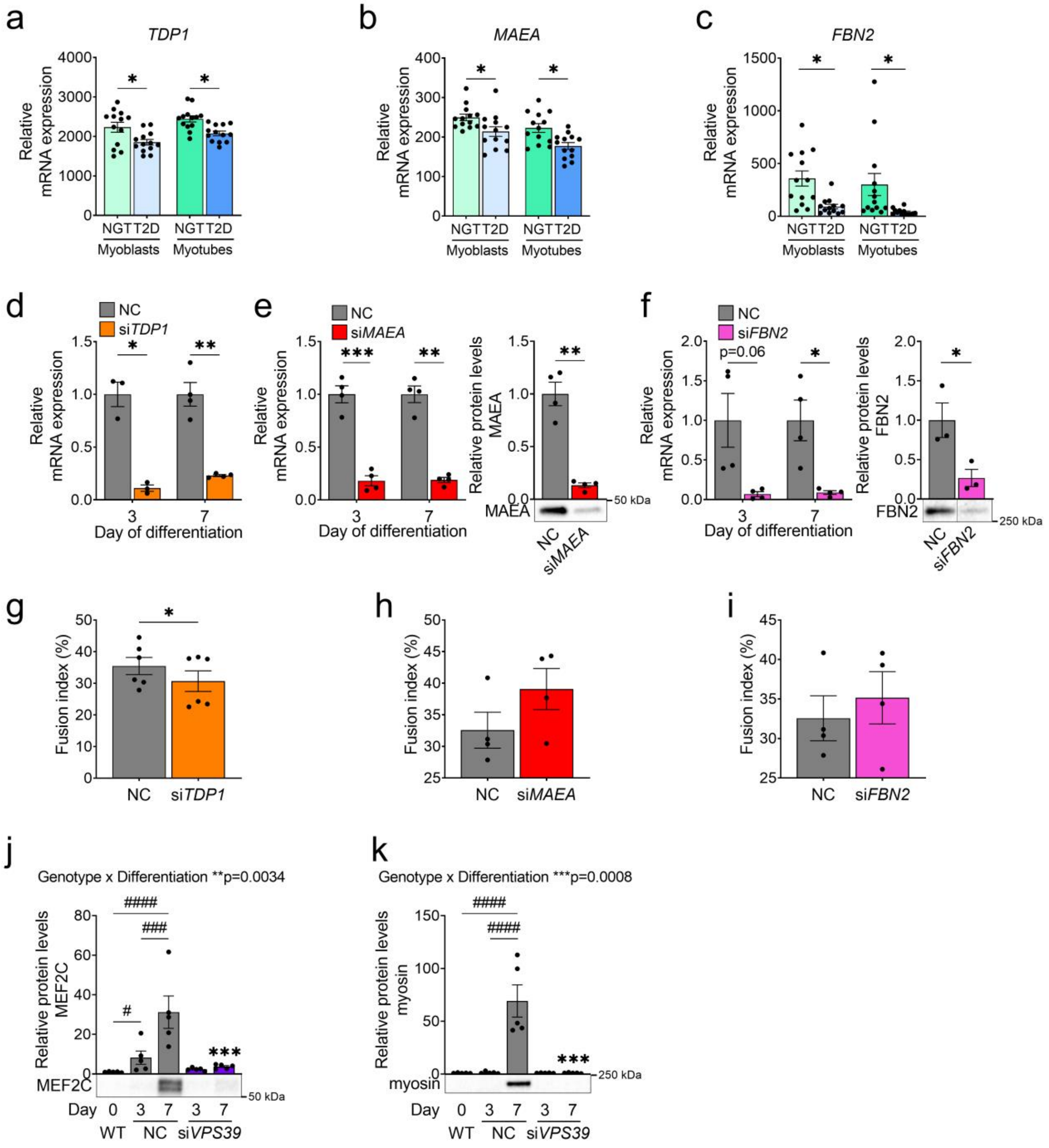

Supplementary Figure 1. Functional follow-up of differentially expressed and methylated genes in human muscle cells from individuals with type 2 diabetes versus controls.

a-c) mRNA expression (microarray) of *TDP1* (a), *MAEA* (b), and *FBN2* (c) in myoblasts and myotubes from individuals with type 2 diabetes (T2D, blue bars) and controls (NGT, normal glucose tolerance, green bars). These genes exhibit differential expression in both myoblasts and myotubes from individuals with T2D vs. NGT. $n = 13$ individuals per group, *q < 0.05 for T2D vs. NGT. For exact q-values see Supplementary Data 1, Sheets A and B.

d-f) Knockdown efficiency (mRNA and protein) of *TDP1* (d, $n = 3$ [Day 3] and $n = 4$ [Day 7] independent experiments), *MAEA* (e, $n = 4$ independent experiments), and *FBN2* (f, $n = 4$ [mRNA] and $n = 3$ [protein] independent experiments) after siRNA silencing (d, si*TDP1* [orange bars], e, si*MAEA* [red bars], and f, si*FBN2* [pink bars]) throughout differentiation. Negative control (NC, gray bars) at each time point is set to 1. Representative blots are shown. We were not able to validate knockdown of TDP1 on protein level due to technical issues. The samples in which FBN2 protein levels were compared were all run on the same gel but not loaded in adjacent lanes. Therefore, the bands are separated with a line to show that they were cropped. The complete blot is provided in the Source data file. *$p < 0.05$, **$p < 0.01$, ***$p < 0.001$ for siRNA vs. NC. $p = 0.0109$ (d, *TDP1* mRNA: Day 3), $p = 0.0053$ (d, *TDP1* mRNA: Day 7), and $p = 0.0003$ (e, *MAEA* mRNA: Day 3), $p = 0.0022$ (e, *MAEA* mRNA: Day 7), $p = 0.0036$ (e, MAEA protein: Day 7), and $p = 0.057$ (f, *FBN2* mRNA: Day 3), $p = 0.0355$ (f, *FBN2* mRNA: Day 7), $p = 0.0237$ (f, FBN2 protein: Day 7).

g-i) Assessment of myotube formation (fusion index) at day 7 of differentiation in si*TDP1* (g, orange bars, $n = 6$ independent experiments), si*MAEA* (h, red bars, $n = 4$ independent experiments) and si*FBN2* (i, pink bars, $n = 4$ independent experiments) and NC (gray bars). *$p < 0.05$ for siRNA vs. NC. $p = 0.0104$ (g, si*TDP1*).

j-k) Protein levels (Western blot) of MEF2C (j) and skeletal myosin (k) in VPS39-silenced muscle cells (si*VPS39*, purple bars) and NC (gray bars) at days 0, 3 and 7 of differentiation. $n = 5$ independent experiments. Wild-type (WT) cells at day 0 are set to 1. Representative blots are shown. #$q < 0.05$, ###q <

0.001, ####*q* < 0.0001 for comparisons between time points within each genotype, and ****q* < 0.001 for si*VPS39* vs. NC at each time point. For exact *q*-values see Supplementary Table 1.

Bars represent mean values and error bars display SEM. Statistical significance determined by linear regression adjusted for age, BMI and sex for T2D vs. NGT (a-c). Statistical significance determined by paired two-tailed t-test (d-i). The effects of genotype (NC and si*VPS39*) and differentiation (Day 0, 3 and 7) stated above the graphs was calculated with repeated measurements two-way ANOVA (j-k). *p*-values were adjusted for multiple comparisons with false discovery rate (FDR) analysis (a-c, j-k).

SUPPLEMENTARY FIGURE 2

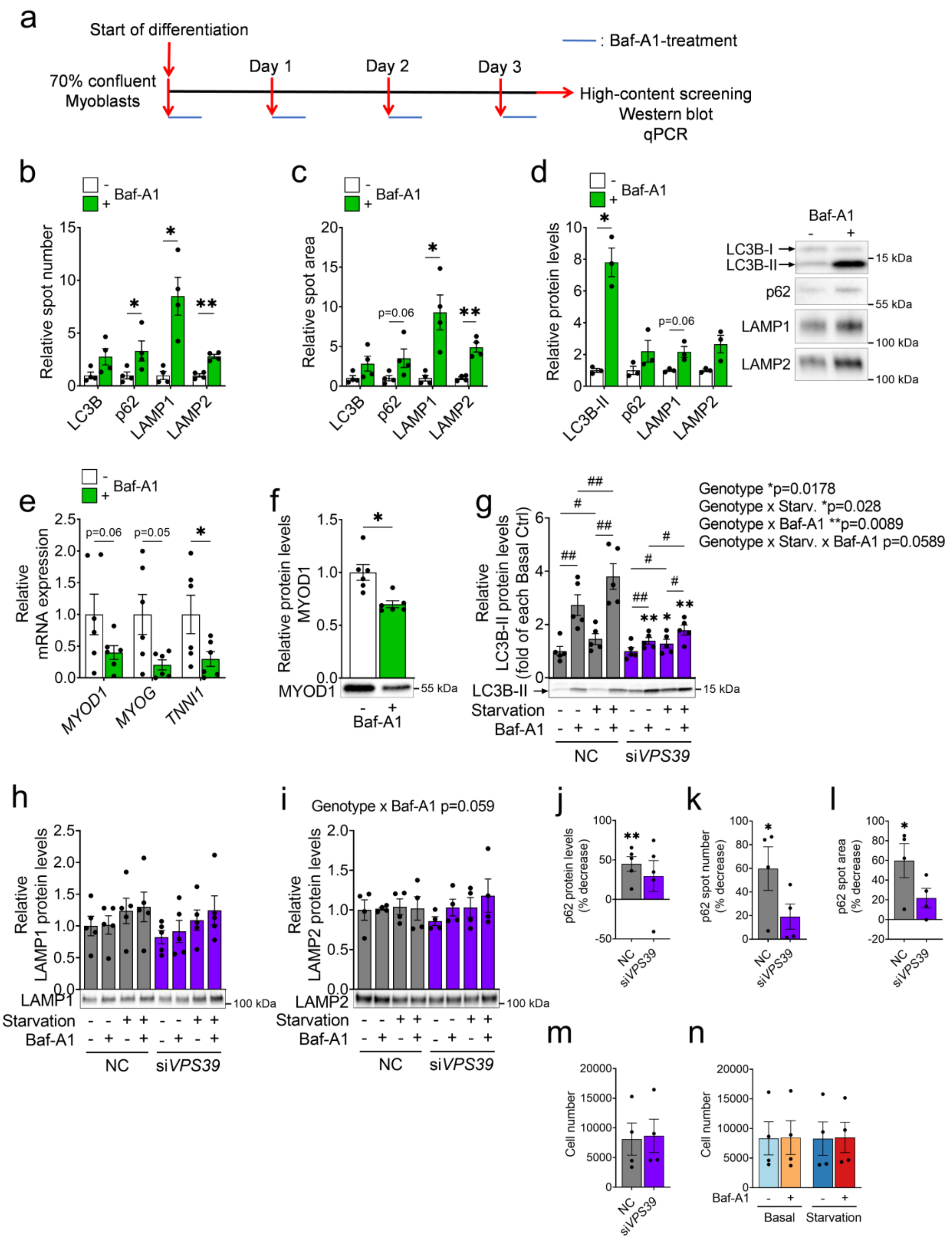

Supplementary Figure 2. Bafilomycin A1 treatment alters autophagy and impairs the expression of myogenic factors in human muscle cells, as well as altered autophagic flux measurements in VPS39-silenced cells.

a) Study design for Bafilomycin-A1 (Baf-A1) treatment of human myoblasts (100 nM, 3 h per day) at days 0-3 of differentiation.

b-c) High-content screening (HCS) analysis using spot detection application to identify autophagy markers LC3B, p62, LAMP1 and LAMP2 in Baf-A1-treated (+, green bars) and vehicle-treated (-, white bars) myoblasts at day 3 of differentiation. $n = 4$ independent experiments. Graphs show relative number of detected spots per cell (b) and area per spot (c) for each marker. Vehicle-treated cells (-) are set to 1. *$p < 0.05$, **$p < 0.01$ for Baf-A1 vs. vehicle. $p = 0.0348$ (b, p62), $p = 0.0181$ (b, LAMP1), $p = 0.0035$ (b, LAMP2), and $p = 0.0555$ (c, p62), $p = 0.0262$ (c, LAMP1), $p = 0.008$ (c, LAMP2).

d) Protein levels (Western blot) of LC3B-II, p62, LAMP1 and LAMP2 in Baf-A1-treated (+, green bars) and vehicle-treated (-, white bars) myoblasts at day 3 of differentiation. $n = 3$ independent experiments. Vehicle-treated cells (-) are set to 1. Representative blots are shown. *$p < 0.05$ for Baf-A1 vs. vehicle. $P = 0.0198$ (LC3B-II), $p = 0.0595$ (LAMP1).

e-f) mRNA expression (qPCR) of *MYOD1*, *MYOG* and *TNNI1* (e), and protein levels (Western blot) of MYOD1 (f) in Baf-A1-treated (+, green bars) and vehicle-treated (-, white bars) myoblasts at day 3 of differentiation. $n = 6$ independent experiments. Vehicle-treated cells (-) are set to 1. Representative blot is shown. *$p < 0.05$ for Baf-A1 vs. vehicle. $p = 0.0577$ (e, *MYOD1* mRNA), $p = 0.0539$ (e, *MYOG* mRNA), $p = 0.0421$ (e, *TNNI1* mRNA), and $p = 0.0206$ (f, MYOD1 protein).

g-l) Autophagic flux measurements in VPS39-silenced (si*VPS39*, purple bars) and negative control (NC, gray bars) myoblasts at day 3 of differentiation in both the basal state and after starvation (3 h) to induce autophagy, and in the absence or presence of the lysosomal inhibitor Bafilomycin A1 (Baf-A1, 100 nM). (g) Protein levels (Western blot) of LC3B-II used to calculate autophagic flux (Figure 3g). $n = 5$ independent experiments. Cells in the basal, vehicle-treated state for each genotype are set to 1. Representative blot is shown. #$q < 0.05$, ##$q < 0.01$ for comparisons between treatments within each

genotype, and *$q < 0.05$, **$q < 0.01$ for si*VPS39* vs. NC for each treatment. For exact *q*-values see Supplementary Table 1. Starv., starvation. (h-i) Protein levels (Western blot) of LAMP1 (h, $n = 5$ independent experiments) and LAMP2 (i, $n = 4$ independent experiments). NC in the basal, vehicle-treated state is set to 1. Representative blots are shown. (j-l) Decrease (%) in p62 protein levels (j, $n = 5$ independent experiments), spot number (k, $n = 4$ independent experiments) and spot area (l, $n = 4$ independent experiments) in response to starvation relative basal, vehicle-treated cells. *$p < 0.05$, **$p < 0.01$. $p = 0.0083$ (j, NC), and $p = 0.0482$ (k, NC), and $p = 0.0402$ (l, NC).

m-n) Cell number (DAPI-stained objects) counted with the HCS assay in the autophagic flux measurements (Figure 3h-p). Mean for all wells treated with (m) si*VPS39* (purple bar) or NC (gray bar), and (n) in response to the different treatments (Basal/vehicle(-) [light blue bar], Basal/Baf-A1(+) [orange bar], Starvation/vehicle(-) [dark blue bar], Starvation/Baf-A1(+) [red bar]). $n = 4$ independent experiments.

Bars represent mean values and error bars display SEM (b-n). Statistical significance determined by paired two-tailed *t*-test (b-f), or one-sample *t*-test comparing the mean of each group (NC or si*VPS39*) to “0” (j-l). The effects of genotype (NC or si*VPS39*), starvation and Baf-A1 stated by the graphs were calculated with repeated measurements three-way ANOVA (g-i). *p*-values were adjusted for multiple comparisons with false discovery rate (FDR) analysis (g).

SUPPLEMENTARY FIGURE 3

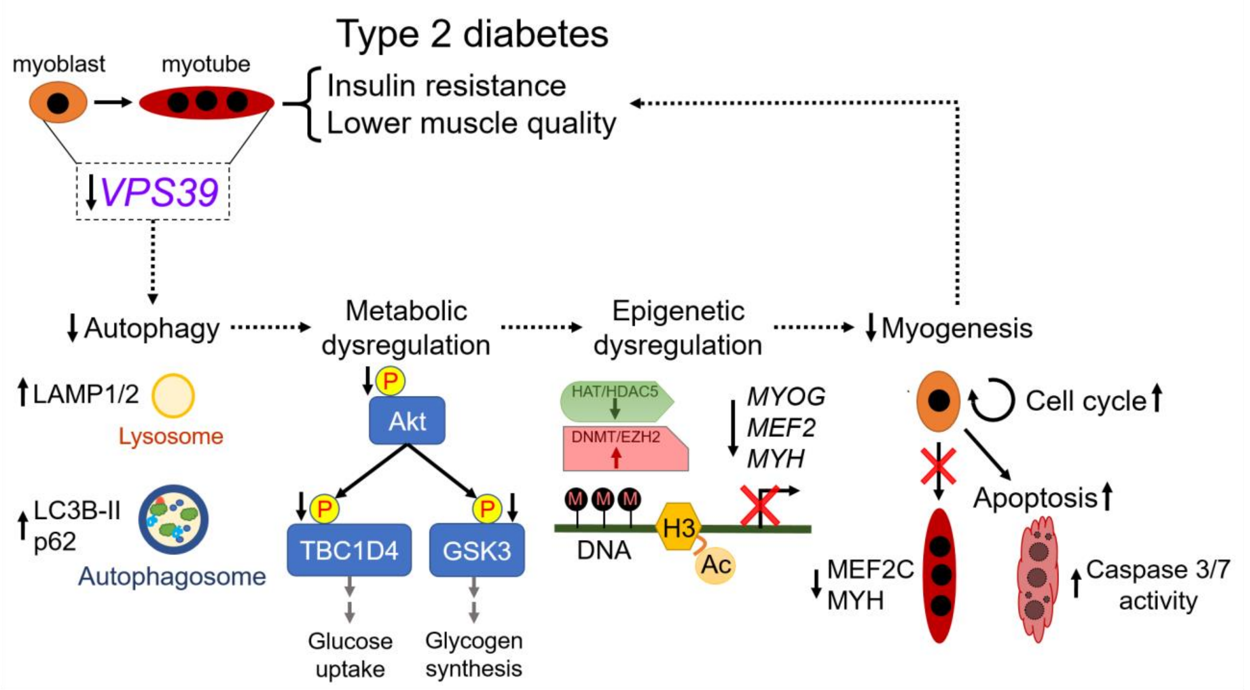

Supplementary Figure 3. Reduced expression of VPS39 impairs autophagy, alters the epigenome and negatively affects myogenesis.

Schematic presentation of the hypothesis that *VPS39 expression is reduced in myoblasts and myotubes from individuals with T2D, which ultimately impacts muscle regeneration and/or growth, and exacerbates the risk of insulin resistance*. The presented data suggest the following molecular mechanism; silencing of VPS39 in human myoblasts is associated with impaired autophagic flux, reduced activation of insulin-regulated metabolic signaling pathways, altered expression and activity of epigenetic enzymes, and changes in DNA methylation. Consequently, the expression of myogenic transcription factors and muscle-specific genes is reduced and myoblasts proliferate, or undergo apoptosis, instead of differentiating into myotubes. Although we suggest an order of events, whereby impaired autophagy alters the metabolic state causing an altered epigenome and in turn impaired myogenesis, it is possible that the progression from VPS39-deficiency to impaired myogenesis is slightly different. Connected processes where causality was not proven directly are depicted by dotted lines. Gray arrows indicate known processes but that weren't studied directly. Akt (PKB), RAC-alpha serine/threonine protein kinase (protein kinase B); DNMT, DNA methyltransferase; EZH2, histone lysine N-methyltransferase EZH2; GSK3, glycogen synthase kinase 3; HAT, histone acetyltransferase; HDAC5, histone deacetylase 5; LAMP, lysosome-associated membrane glycoprotein; LC3B, microtubule-associated proteins 1A/1B light chain 3B; M, methylation; MEF, myocyte-specific enhancer factor; MYH, myosin; MYOG, myogenin; p62 (SQSTM1), ubiquitin-binding protein p62 (sequestosome 1); TBC1D4 (AS160), TBC1 domain family member 4 (Akt substrate of 160 kDa); VPS39, Vam6/Vps39-like protein

SUPPLEMENTARY FIGURE 4

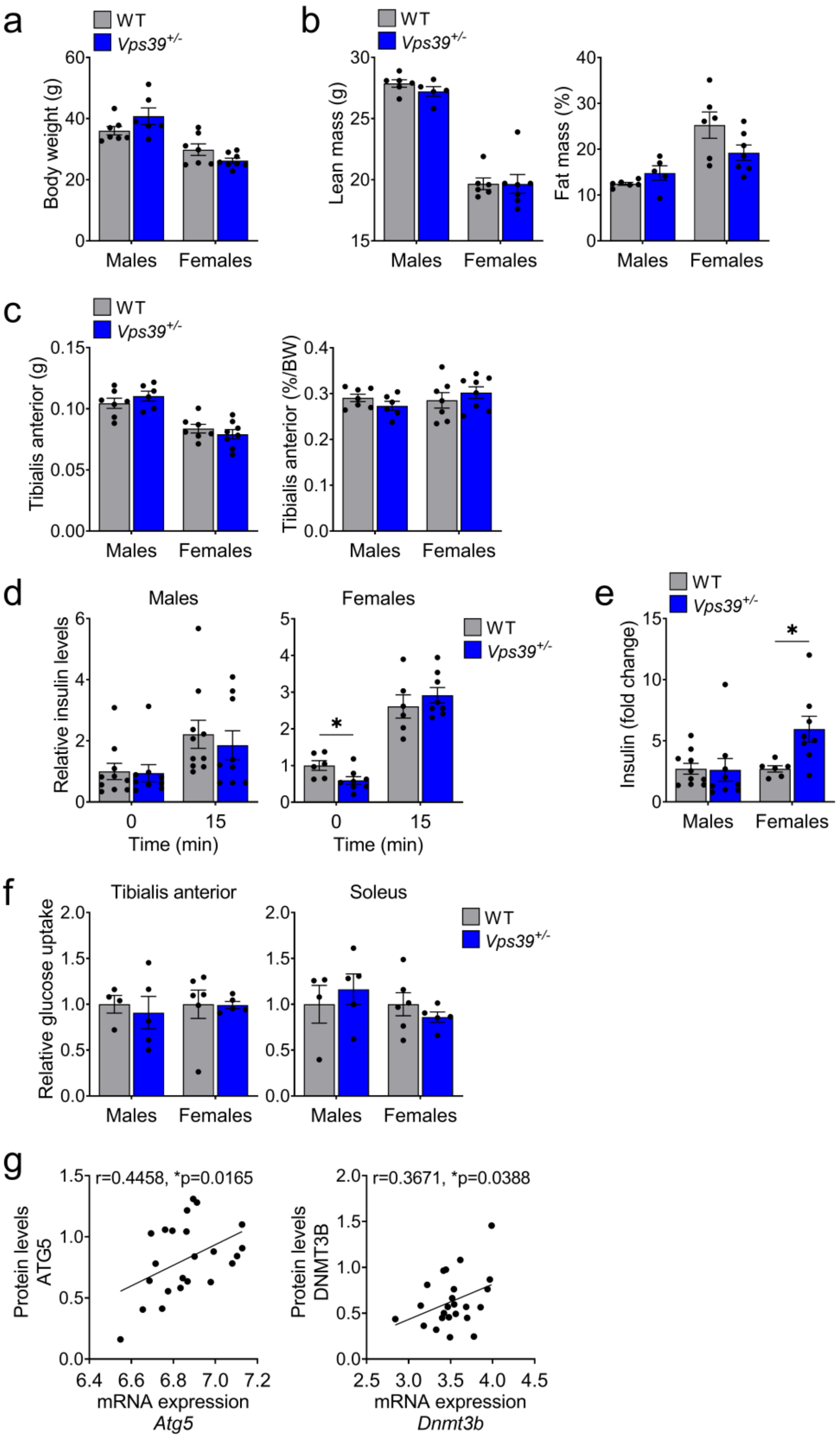

Supplementary Figure 4. Body composition and metabolic characteristics of *Vps39*$^{+/-}$ mice in comparison to wild-type mice.

a-c) Body composition of wild-type mice (WT, gray bars) and *Vps39*$^{+/-}$ heterozygous mice (blue bars). Mice were 4-5 months old. (a) Body weight in grams. n = 7 WT males and 7 WT females, and n = 6 *Vps39*$^{+/-}$ males and 8 *Vps39*$^{+/-}$ females. (b) Whole body lean mass in grams (left panel), and fat mass in % (right panel) determined by DEXA scan. n = 6 WT males and 6 WT females, and n = 5 *Vps39*$^{+/-}$ males and 7 *Vps39*$^{+/-}$ females. (c) Absolute (left panel, in grams) and relative (right panel, %/BW, body weight) muscle mass of tibialis anterior. n = 7 WT males and 7 WT females, and n = 6 *Vps39*$^{+/-}$ males and 8 *Vps39*$^{+/-}$ females.

d-e) Insulin levels during the oral glucose tolerance test (OGTT) presented in Figure 5b in male (n = 10 WT and n = 9 *Vps39*$^{+/-}$) and female (n = 6 WT and n = 8 *Vps39*$^{+/-}$) mice. WT mice (gray bars) and *Vps39*$^{+/-}$ heterozygous mice (blue bars). (d) Relative insulin levels at baseline (0 min) and 15 min after a glucose challenge in male (left panel) and female (right panel) mice. WT mice at baseline are set to 1. *$p < 0.05$ for *Vps39*$^{+/-}$ vs. WT. $p = 0.0298$ (females, 0 min). (e) Fold change in insulin levels during the first 15 min of the OGTT (insulin levels at 15 min relative 0 min). *$p < 0.05$ for *Vps39*$^{+/-}$ vs. WT. $p = 0.0229$ (females).

f) Relative glucose uptake in the tibialis anterior (left panel) and soleus (right panel) muscles during 45 min after an oral glucose load. n = 4 WT males and 6 WT females (gray bars), and n = 5 *Vps39*$^{+/-}$ males and 5 *Vps39*$^{+/-}$ females (blue bars). Glucose uptake was measured using 2-[1,2-^{3}H(N)]-Deoxy-D-glucose tracer and normalized to tissue weight. WT mice are set to 1.

g) Correlations between ATG5 (left panel) and DNMT3B (right panel) mRNA (microarray) and protein levels (Western blot) in tibialis anterior muscles. n = 6 WT males, and 5 WT females (for ATG5) or 6 WT females (for DNMT3B), and n = 6 *Vps39*$^{+/-}$ males and 6 *Vps39*$^{+/-}$ females.

Bars represent mean values and error bars display SEM (a-f). Statistical significance determined by unpaired two-tailed t-test (d-e), or one-tailed Pearson correlation test (g).

SUPPLEMENTARY FIGURE 5

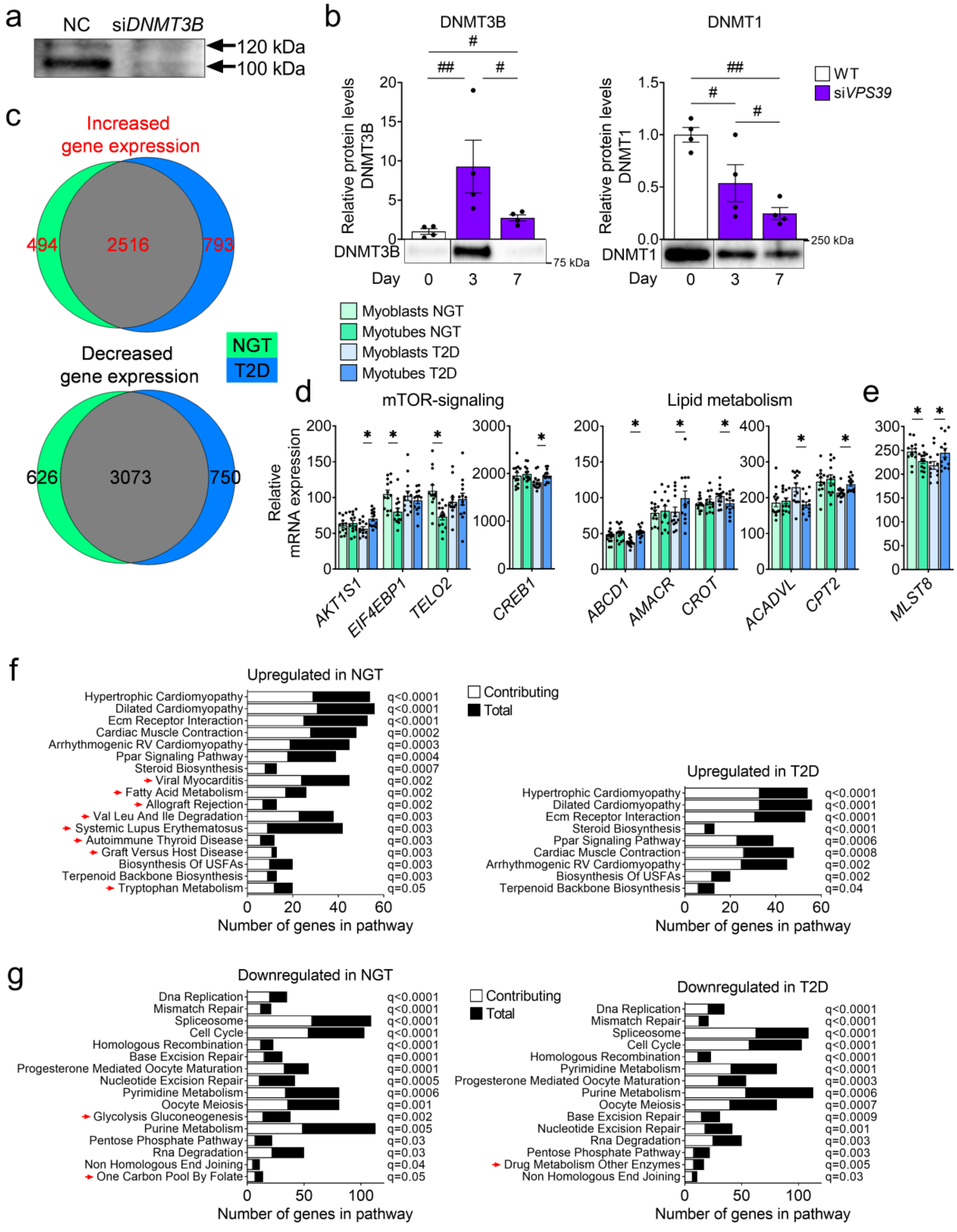

Supplementary Figure 5. Altered DNA methylation and gene expression patterns during differentiation of myoblasts from individuals with type 2 diabetes and controls.

a) Western blot analysis of DNMT3B protein after siRNA-mediated DNMT3B knockdown (si*DNMT3B*) and negative control (NC). Myoblasts were transfected at the start of differentiation and DNMT3B protein was analyzed on day 5 of differentiation. $n = 1$ independent experiment.

b) DNMT3B (left panel) and DNMT1 (right panel) protein levels (Western blot) in wild-type (WT, white bars) myoblasts at day 0 and VPS39-silenced (si*VPS39*, purple bars) muscle cells at days 3 and 7 of differentiation. $n = 4$ independent experiments. WT cells at day 0 are set to 1. Representative blots are shown. The paired samples in which DNMT3B or DNMT1 protein levels were compared were all run on the same gel but not loaded in adjacent lanes. Therefore, the bands are separated with a line to show that they were cropped. The complete blot is provided in the Source data file. #$q < 0.05$, ##$q < 0.01$ for comparisons between time points. $q = 0.005$ (DNMT3B: Day 0 vs. 3), $q = 0.0407$ (DNMT3B: Day 0 vs. 7), $q = 0.048$ (DNMT3B: Day 3 vs. 7), and $q = 0.0371$ (DNMT1: Day 0 vs. 3), $q = 0.0049$ (DNMT1: Day 0 vs. 7), $q = 0.0403$ (DNMT1: Day 3 vs. 7).

c) Venn diagrams showing the number of overlapping and unique genes that were differentially expressed in myoblasts vs. myotubes from individuals with type 2 diabetes (T2D, blue) and controls (NGT, normal glucose tolerance, green). Genes with increased expression (top panel, red text) and decreased expression (bottom panel, black text).

d-e) Gene expression (microarray) in myoblasts and myotubes for a selection of genes (mTOR-signaling and lipid metabolism) that changed expression in either individuals with T2D (blue bars) or NGT (green bars) (d), and *MLST8* which changed expression in opposite direction in individuals with T2D and NGT (e). $n = 13$ individuals per group. *$q < 0.05$ for myotubes vs. myoblasts within each group (NGT or T2D). For exact *q*-values see Supplementary Data 1, Sheets C and D.

f-g) Significantly enriched gene sets (FDR < 5%) based on GSEA of microarray expression data comparing myoblasts vs. myotubes from individuals with T2D (right panels) and NGT (left panels). Gene sets that were upregulated (f) and downregulated (g) before vs. after muscle cell differentiation. Bars

represent the number of differentially expressed genes contributing to each gene set (white bars), and the total number of genes in each gene set (black bars). Red arrows indicate gene sets regulated only in one of the groups (NGT or T2D). RV, right ventricular; Val, valine; Leu, leucine; Ile, isoleucine; USFA, unsaturated fatty acid.

Bars represent mean values and error bars display SEM (b, d-e). Statistical significance determined by repeated measures one-way ANOVA (b). DNMT3B and DNMT1 protein values were log2-transformed before statistical analysis. Non-logarithmic values are presented in the graphs (b). Statistical significance determined by Wilcoxon signed-rank test for myoblasts vs. myotubes from the same individuals (d-e). *p*-values were adjusted for multiple comparisons with false discovery rate (FDR) analysis (b, d-g).

SUPPLEMENTARY FIGURE 6

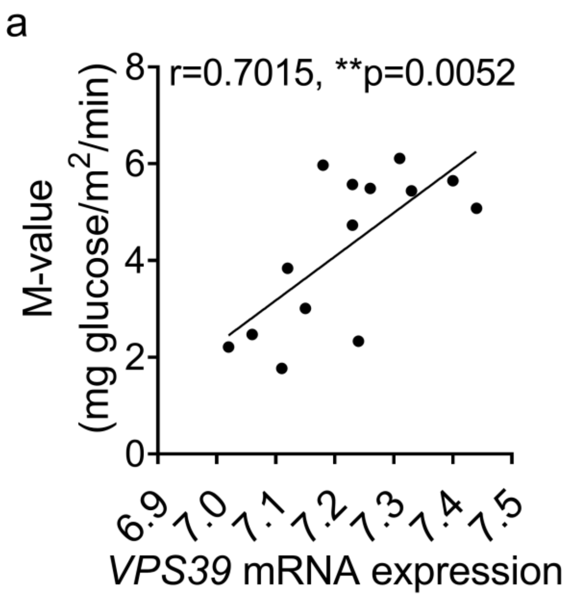

Supplementary Figure 6. *VPS39* mRNA expression in human muscle biopsies correlate positively with glucose uptake, and is nominally reduced in individuals with type 2 diabetes versus controls.

a) Correlation between *VPS39* mRNA expression (microarray) in skeletal muscle and glucose uptake measured during a hyperinsulinemic euglycemic clamp (M-value, mg glucose/m^2/min) in n = 14 individuals with type 2 diabetes (T2D).

b) *VPS39* mRNA expression in skeletal muscle from n = 14 individuals with T2D and n = 17 controls (NGT, normal glucose tolerance). Box represents 25th to 75th percentiles and the box line is plotted at the median. Whiskers represent min and max values.

Statistical significance determined by two-tailed Pearson correlation test (a), or one-tailed Mann-Whitney test (b).

SUPPLEMENTARY TABLES

Supplementary Table 1. Exact *p*- and *q*-values for statistical analyses in Figures 3e-f, 3h-i, 3k-l, 3n-o, 4a-c, 4i, and Supplementary Figures 1j-k, 2g.

**Figure 3e: LC3B-II protein levels**		
**Source of variation**	**% of total variation**	***p*-value**
Genotype	22.26	0.0034
Starvation	9.31	0.0111
Baf-A1	34.87	0.0002
**Comparison**	***p*-value**	***q*-value (FDR-adjusted *p*-value)**
NC:Basal Ctrl vs. si*VPS39*:Basal Ctrl	0.0011	0.0019
NC:Basal Baf-A1 vs. si*VPS39*:Basal Baf-A1	0.0045	0.0047
NC:Starvation Ctrl vs. si*VPS39*:Starvation Ctrl	0.0013	0.0019
NC:Starvation Baf-A1 vs. si*VPS39*:Starvation Baf-A1	0.1592	0.0696
NC:Basal Ctrl vs. NC:Basal Baf-A1	0.0056	0.0049
NC:Starvation Ctrl vs. NC:Starvation Baf-A1	0.0014	0.0019
si*VPS39*:Basal Ctrl vs. si*VPS39*:Basal Baf-A1	0.001	0.0019
si*VPS39*:Starvation Ctrl vs. si*VPS39*:Starvation Baf-A1	0.065	0.0367
NC:Basal Ctrl vs. NC:Starvation Ctrl	0.0771	0.0368
NC:Basal Baf-A1 vs. NC:Starvation Baf-A1	0.0068	0.0051
si*VPS39*:Basal Ctrl vs. si*VPS39*:Starvation Ctrl	0.0614	0.0367
si*VPS39*:Basal Baf-A1 vs. si*VPS39*:Starvation Baf-A1	0.07	0.0367

**Figure 3f: p62 protein levels**		
**Source of variation**	**% of total variation**	***p*-value**
Genotype	45.41	0.0154
Baf-A1	15.96	0.0188
Genotype x Baf-A1	10.18	0.0087
**Comparison**	***p*-value**	***q*-value (FDR-adjusted *p*-value)**
NC:Basal Ctrl vs. si*VPS39*:Basal Ctrl	0.0403	0.0423
NC:Basal Baf-A1 vs. si*VPS39*:Basal Baf-A1	0.0062	0.0236
NC:Starvation Ctrl vs. si*VPS39*:Starvation Ctrl	0.0294	0.0361
NC:Starvation Baf-A1 vs. si*VPS39*:Starvation Baf-A1	0.016	0.0236
NC:Basal Ctrl vs. NC:Basal Baf-A1	0.0734	0.06
NC:Starvation Ctrl vs. NC:Starvation Baf-A1	0.0477	0.0438
si*VPS39*:Basal Ctrl vs. si*VPS39*:Basal Baf-A1	0.0064	0.0236
si*VPS39*:Starvation Ctrl vs. si*VPS39*:Starvation Baf-A1	0.0111	0.0236
NC:Basal Ctrl vs. NC:Starvation Ctrl	0.0161	0.0236

**Figure 3h: p62 spot number**		
**Source of variation**	**% of total variation**	***p*-value**
Genotype	22.57	0.0243
Baf-A1	6.65	0.0453
**Comparison**	***p*-value**	***q*-value (FDR-adjusted *p*-value)**
NC:Basal Ctrl vs. si*VPS39*:Basal Ctrl	0.067	0.0603

NC:Basal Baf-A1 vs. si*VPS39*:Basal Baf-A1	0.0177	0.0186
NC:Starvation Ctrl vs. si*VPS39*:Starvation Ctrl	0.0066	0.0129
NC:Starvation Baf-A1 vs. si*VPS39*:Starvation Baf-A1	0.0068	0.0129
NC:Starvation Ctrl vs. NC:Starvation Baf-A1	0.0098	0.0129
si*VPS39*:Starvation Ctrl vs. si*VPS39*:Starvation Baf-A1	0.0103	0.0129
NC:Basal Ctrl vs. NC:Starvation Ctrl	0.0089	0.0129

**Figure 3i: p62 spot area**		
**Source of variation**	**% of total variation**	***p*-value**
Genotype	19.53	0.0516
**Comparison**	***p*-value**	***q*-value (FDR-adjusted *p*-value)**
NC:Basal Baf-A1 vs. si*VPS39*:Basal Baf-A1	0.0261	0.0743
NC:Starvation Ctrl vs. si*VPS39*:Starvation Ctrl	0.0295	0.0743
NC:Starvation Baf-A1 vs. si*VPS39*:Starvation Baf-A1	0.0087	0.0674
si*VPS39*:Starvation Ctrl vs. si*VPS39*:Starvation Baf-A1	0.0107	0.0674
NC:Basal Ctrl vs. NC:Starvation Ctrl	0.0195	0.0743

**Figure 3k: LAMP1 spot number**		
**Source of variation**	**% of total variation**	***p*-value**
Genotype	14.26	0.0005
Starvation	9.02	0.0359
Baf-A1	22.84	0.048

**Comparison**	***p*-value**	***q*-value (FDR-adjusted *p*-value)**
NC:Basal Ctrl vs. si*VPS39*:Basal Ctrl	0.001	0.0056
NC:Basal Baf-A1 vs. si*VPS39*:Basal Baf-A1	0.0018	0.0056
NC:Starvation Baf-A1 vs. si*VPS39*:Starvation Baf-A1	0.0177	0.0186
NC:Basal Ctrl vs. NC:Basal Baf-A1	0.004	0.0085
si*VPS39*:Basal Ctrl vs. si*VPS39*:Basal Baf-A1	0.0062	0.0091
si*VPS39*:Starvation Ctrl vs. si*VPS39*:Starvation Baf-A1	0.0424	0.0334
NC:Basal Baf-A1 vs. NC:Starvation Baf-A1	0.0416	0.0334
si*VPS39*:Basal Ctrl vs. si*VPS39*:Starvation Ctrl	0.0527	0.0369
si*VPS39*:Basal Baf-A1 vs. si*VPS39*:Starvation Baf-A1	0.0072	0.0091

**Figure 3l: LAMP1 spot area**		
**Source of variation**	**% of total variation**	***p*-value**
Genotype	19.87	0.0017
Starvation	7.78	0.0331
Baf-A1	19.92	0.0635
**Comparison**	***p*-value**	***q*-value (FDR-adjusted *p*-value)**
NC:Basal Ctrl vs. si*VPS39*:Basal Ctrl	0.0024	0.0038
NC:Basal Baf-A1 vs. si*VPS39*:Basal Baf-A1	0.0001	0.0009
NC:Starvation Baf-A1 vs. si*VPS39*:Starvation Baf-A1	0.0236	0.0248
NC:Basal Ctrl vs. NC:Basal Baf-A1	0.0202	0.0248

si*VPS39*:Basal Ctrl vs. si*VPS39*:Basal Baf-A1	0.0017	0.0036
si*VPS39*:Basal Baf-A1 vs. si*VPS39*:Starvation Baf-A1	0.0016	0.0036

**Figure 3n: LAMP2 spot number**		
**Source of variation**	**% of total variation**	***p*-value**
Genotype	39.89	0.0011
Baf-A1	9.85	0.0536
Genotype x Baf-A1	4.75	0.005
**Comparison**	***p*-value**	***q*-value (FDR-adjusted *p*-value)**
NC:Basal Ctrl vs. si*VPS39*:Basal Ctrl	0.0042	0.0066
NC:Basal Baf-A1 vs. si*VPS39*:Basal Baf-A1	0.0002	0.0007
NC:Starvation Ctrl vs. si*VPS39*:Starvation Ctrl	0.0145	0.0183
NC:Starvation Baf-A1 vs. si*VPS39*:Starvation Baf-A1	0.0002	0.0007
si*VPS39*:Basal Ctrl vs. si*VPS39*:Basal Baf-A1	0.0766	0.0603
si*VPS39*:Starvation Ctrl vs. si*VPS39*:Starvation Baf-A1	0.0024	0.0051
NC:Basal Ctrl vs. NC:Starvation Ctrl	0.0482	0.0434
si*VPS39*:Basal Ctrl vs. si*VPS39*:Starvation Ctrl	0.0193	0.0203

**Figure 3o: LAMP2 spot area**		
**Source of variation**	**% of total variation**	***p*-value**
Genotype	37.67	0.0141
Baf-A1	13.42	0.0109
Genotype x Baf-A1	4.91	0.0064

**Comparison**	***p*-value**	***q*-value (FDR-adjusted *p*-value)**
NC:Basal Ctrl vs. si*VPS39*:Basal Ctrl	0.0092	0.0116
NC:Basal Baf-A1 vs. si*VPS39*:Basal Baf-A1	0.001	0.0033
NC:Starvation Baf-A1 vs. si*VPS39*:Starvation Baf-A1	0.0016	0.0033
si*VPS39*:Basal Ctrl vs. si*VPS39*:Basal Baf-A1	0.0066	0.0104
si*VPS39*:Starvation Ctrl vs. si*VPS39*:Starvation Baf-A1	0.0005	0.0032
si*VPS39*:Basal Ctrl vs. si*VPS39*:Starvation Ctrl	0.0122	0.0128

**Figure 4a: Akt phosphorylation**		
	**Comparison**	***p*-value**
p-Akt Ser473	NC:Basal vs. NC:Insulin	0.008
	si*VPS39*:Basal vs. si*VPS39*:Insulin	0.0501
	NC:Insulin vs. si*VPS39*:Insulin	0.0295
p-Akt Thr308	NC:Basal vs. NC:Insulin	0.0118
	NC:Insulin vs. si*VPS39*:Insulin	0.0284

**Figure 4b: TBC1D4 phosphorylation**		
**Source of variation**	**% of total variation**	***p*-value**
Insulin	14.92	0.0365
Insulin x Genotype	0.5	0.0408

	Comparison	*p*-value
p-TBC1D4 Thr642	NC:Basal vs. NC:Insulin	0.0016
	si*VPS39*:Basal vs. si*VPS39*:Insulin	0.0005
	NC:Basal vs. si*VPS39*:Basal	0.0026
	NC:Insulin vs. si*VPS39*:Insulin	0.021

**Figure 4c: GSK3 phosphorylation**		
-α Ser21		
**Source of variation**	**% of total variation**	***p*-value**
Insulin	43.81	0.0109
-β Ser9		
**Source of variation**	**% of total variation**	***p*-value**
Insulin	57.62	0.0151
Genotype	5.03	0.0269
	**Comparison**	***p*-value**
p-GSK3-α Ser21	NC:Basal vs. NC:Insulin	0.008
	si*VPS39*:Basal vs. si*VPS39*:Insulin	0.0361
	NC:Insulin vs. si*VPS39*:Insulin	0.055
p-GSK3-β Ser9	NC:Basal vs. NC:Insulin	0.0203
	NC:Insulin vs. si*VPS39*:Insulin	0.0215

Figure 4i: H3 acetylation		
**Source of variation**	**% of total variation**	***p*-value**
Genotype	5.58	0.0128
Differentiation	46.89	0.0346
Genotype x Differentiation	4.2	0.0065
**Comparison**	***p*-value**	***q*-value (FDR-adjusted p-value)**
WT:Day 0 vs.<br>NC:Day 3	9e-06	1.5e-05
WT:Day 0 vs.<br>NC:Day 7	1e-06	4e-06
NC:Day 3 vs.<br>NC:Day 7	0.0158	0.0166
WT:Day 0 vs.<br>si*VPS39*:Day 3	9.1e-05	9.1e-05
WT:Day 0 vs.<br>si*VPS39*:Day 7	1.7e-04	9.1e-05
NC:Day 3 vs.<br>si*VPS39*:Day 3	0.0294	0.0154
NC:Day 7 vs.<br>si*VPS39*:Day 7	2.3e-04	2.4e-04

Supplementary Figure 1j: MEF2C protein levels		
**Source of variation**	**% of total variation**	***p*-value**
Genotype	18.5	0.0368
Differentiation	29.13	0.0015
Genotype x Differentiation	20.94	0.0034
**Comparison**	***p*-value**	***q*-value (FDR-adjusted *p*-value)**
WT:Day 0 vs.<br>NC:Day 3	0.1156	0.0404
WT:Day 0 vs.<br>NC:Day 7	7.7e-05	8.1e-05
NC:Day 3 vs.<br>NC:Day 7	0.0005	0.00026
NC:Day 7 vs.<br>si*VPS39*:Day 7	0.00015	0.00032

Supplementary Figure 1k: Myosin protein levels		
**Source of variation**	**% of total variation**	***p*-value**
Genotype	16.27	0.0103
Differentiation	32.15	0.0009
Genotype x Differentiation	32.06	0.0008
**Comparison**	***p*-value**	***q*-value (FDR-adjusted *p*-value)**
WT:Day 0 vs.<br>NC:Day 7	5.7e-05	3.1e-05
NC:Day 3 vs.<br>NC:Day 7	6e-05	3.1e-05
NC:Day 7 vs.<br>si*VPS39*:Day 7	5.8e-05	0.00012

Supplementary Figure 2g: LC3B-II protein levels		
**Source of variation**	**% of total variation**	***p*-value**
Genotype	17.5	0.0178
Starvation	6.88	0.0262
Baf-A1	34.63	0.0027
Genotype x Starvation	1	0.028
Genotype x Baf-A1	14.22	0.0089
Genotype x Starvation x Baf-A1	0.35	0.0589
**Comparison**	***p*-value**	***q*-value (FDR-adjusted *p*-value)**
NC:Basal Baf-A1 vs. si*VPS39*:Basal Baf-A1	0.0084	0.0074
NC:Starvation Ctrl vs. si*VPS39*:Starvation Ctrl	0.0167	0.0125
NC:Starvation Baf-A1 vs. si*VPS39*:Starvation Baf-A1	0.0083	0.0074
NC:Basal Ctrl vs. NC:Basal Baf-A1	0.0056	0.0074
NC:Starvation Ctrl vs. NC:Starvation Baf-A1	0.0014	0.0037
si*VPS39*:Basal Ctrl vs. si*VPS39*:Basal Baf-A1	0.001	0.0037
si*VPS39*:Starvation Ctrl vs. si*VPS39*:Starvation Baf-A1	0.065	0.0367
NC:Basal Ctrl vs. NC:Starvation Ctrl	0.0771	0.0368
NC:Basal Baf-A1 vs. NC:Starvation Baf-A1	0.0068	0.0074
si*VPS39*:Basal Ctrl vs. si*VPS39*:Starvation Ctrl	0.0614	0.0367
si*VPS39*:Basal Baf-A1 vs. si*VPS39*:Starvation Baf-A1	0.07	0.0367

Supplementary Table 2. List of primers used for qPCR

**TAQMAN ASSAYS (Applied Biosystems)**	
**Target**	**Assay**
FBN2	Hs00266592_m1
MAEA	Hs00169481_m1
TDP1	Hs00217832_m1
VPS39	Hs00385779_m1
PPIA	4326316E-0901011
UNC50	Hs00204093_m1
Vps39	Mm01268602_m1[a]
Gusb	Mm01197698_m1
Ppia	Mm02342430_g1

[a] located at the boundary of *Vps39* exon 1 and 2, and spanning the deleted fragment of exon 2 in *Vps39*$^{+/-}$ mice

**SYBRGREEN PRIMERS (DNA Technology A/S)**	
**Target**	**Primer sequence**
MYOD1	f-5'CACTACAGCGGGCGACTCC<br>r-5'TAGGCGCCTTCGTAGCAG
MYOG	f-5'GCTCAGCTCCCTCAACCA<br>r-5'GCTGTGAGAGCTGCATTCG
TNNI1	f-5'GGCCAACCTCAAGTCTGTG<br>r-5'AGACATGGCCTCCACGTT

f, forward; r, reverse

Supplementary Table 3. List of antibodies used for Western blot and immunocytochemistry

WESTERN BLOT				
**Target**	**Primary Antibody**	**Dilution**	**Blocking**	**Secondary antibody**
DNMT3B (mouse)	SCBT Cat# sc-376043, RRID:AB_10988201)	1:200 in 5% BSA/TBST	5% milk/TBST	Goat anti-mouse IgG (HRP) Bio-Rad Cat# 170-6516, RRID:AB_11125547 1:5000 in 5% milk/TBST
EZH2	CST Cat# 3147, RRID:AB_10694383	1:1000 in 5% BSA/TBST	5% milk/TBST	
LAMP1	DSHB Cat# H4A3, RRID:AB_2296838	0.3 μg/ml in 5% BSA/TBST	5% milk/TBST	
LAMP2	DSHB Cat# H4B4, RRID:AB_528129	0.3 μg/ml in 5% BSA/TBST	5% milk/TBST	
MYOD	SCBT Cat# sc-377460, RRID:AB_2813894	1:200 in 5% BSA/TBST	5% milk/TBST	
Myosin	Sigma-Aldrich Cat# M4276, RRID:AB_477190	1:1000 in 5% BSA/TBST	5% milk/TBST	
VPS39	SCBT Cat# sc-514762, RRID:AB_2687985	1:200 in 5% BSA/TBST	5% milk/TBST	
Akt	CST Cat# 9272, RRID:AB_329827	1:1000 in 5% BSA/TBST	5% milk/TBST	Goat anti-rabbit IgG (HRP) CST Cat# 7074, RRID:AB_2099233 1:10,000 in 5% milk/TBST
p-Akt Thr308	CST Cat# 9275, RRID:AB_329828	1:1000 in 5% BSA/TBST	5% milk/TBST	
p-Akt Ser473	CST Cat# 9271, RRID:AB_329825	1:1000 in 5% BSA/TBST	5% milk/TBST	
ATG5	CST Cat# 12994, RRID:AB_2630393	1:1000 in 5% BSA/TBST	5% milk/TBST	
DNMT1	NB Cat# NB100-264, RRID:AB_10000772	1:1000 in 5% BSA/TBST	5% milk/TBST	

DNMT3A	Sigma-Aldrich Cat# HPA026588, RRID:AB_1847812	1:250 in 5% BSA/TBST	5% milk/TBST	
DNMT3B (human)	Sigma-Aldrich Cat#HPA001595, RRID:AB_1847814	1:250 in 5% BSA/TBST	5% milk/TBST	
FBN2	NB Cat# NBP1-88169, RRID:AB_11034933	0.4 µg/ml in 5% BSA/TBST	5% milk/TBST	
GSK3α/β	Thermo Fisher Scientific Cat# 44-610, RRID:AB_2533693	1:1000 in 5% BSA/TBST	5% milk/TBST	
p-GSK3α/β Ser21/Ser9	CST Cat# 9331, RRID:AB_329830	1:1000 in 5% BSA/TBST	5% milk/TBST	
Histone H3	Abcam Cat# ab1791, RRID:AB_302613	1:4000 in 5% BSA/TBST	5% BSA/TBST	
acetylated-Histone H3	Millipore Cat# 06-599, RRID:AB_2115283	1:500 in 5% BSA/TBST	5% milk/TBST	
HAT1	SCBT Cat# sc-366092, discontinued	1:200 in 5% BSA/TBST	5% milk/TBST	
HDAC4	Abcam Cat# ab12172, RRID:AB_298904	1:1000 in 5% BSA/TBST	5% milk/TBST	
HDAC5	CST Cat# 20458, RRID:AB_2713973	1:1000 in 5% BSA/TBST	5% milk/TBST	
LC3B	NB Cat# NB100-2220, RRID:AB_10003146	1:1000 in 5% BSA/TBST	5% milk/TBST	
MEF2C	Abcam Cat# ab211493, RRID:AB_2864417	1:1000 in 5% BSA/TBST	5% milk/TBST	

p300	SCBT Cat# sc-584, RRID:AB_2293429	1:200 in 5% BSA/TBST	5% milk/TBST	
p62	Abcam Cat# ab91526, RRID:AB_2050336	1:1000 in 5% BSA/TBST	5% milk/TBST	
TBC1D4 (AS160)	Millipore Cat# 07-741, RRID:AB_492639	1:1000 in 5% BSA/TBST	5% milk/TBST	
p-TBC1D4 (AS160) Thr642	Thermo Fisher Scientific Cat# 44-1071G, RRID:AB_2533564	1:1000 in 5% BSA/TBST	5% milk/TBST	
MAEA	R&D Systems Cat# AF7288, RRID:AB_10971438	1 µg/ml in 5% milk/TBST	5% milk/TBST	Rabbit anti-sheep IgG (HRP) P0163, Dako 1:2,500 in 5% milk/TBST

**IMMUNOCYTOCHEMISTRY (FUSION INDEX AND HCS)**				
**Target**	**Primary Antibody**	**Dilution**	**Blocking**	**Secondary antibody**
LAMP1	DSHB Cat# H4A3, RRID:AB_2296838	3 µg/ml in 5% goat serum/PBS	5% goat serum/PBS	Goat anti-mouse IgG (Cy2) Jackson ImmunoResearch Labs Cat# 115-225-146, RRID:AB_2307343 1:400 in 5% goat serum/PBS
LAMP2	DSHB Cat# H4B4, RRID:AB_528129	3 µg/ml in 5% goat serum/PBS	5% goat serum/PBS	
Myosin	DSHB Cat# MF 20, RRID:AB_2147781	1:50 in HBSS	3% BSA/PBS	
LC3B	NB Cat# NB100-2220, RRID:AB_10003146	1:1000 in 5% goat serum/PBS	5% goat serum/PBS	Goat anti-rabbit IgG (Cy3) Jackson ImmunoResearch

p62	Abcam Cat# ab91526, RRID:AB_2050336	1:500 in 5% goat serum/PBS	5% goat serum/PBS	Labs Cat# 111-165-144, RRID:AB_2338006 1:400 in 5% goat serum/PBS

CST, Cell Signaling Technology; DSHB, Developmental Studies Hybridoma Bank; NB, Novus Biologicals; SCBT, Santa Cruz Biotechnology

BSA, Bovine serum albumin; HBSS, Hank's balanced salt solution; HRP, horseradish peroxidase; PBS, phosphate buffered saline; TBST, Tris buffered saline with 0.05 % [w/v] Tween-20.

SUPPLEMENTARY REFERENCES

1 Bibikova, M. *et al.* High density DNA methylation array with single CpG site resolution. *Genomics* **98**, 288-295, doi:10.1016/j.ygeno.2011.07.007 (2011).

2 Davegardh, C. *et al.* Abnormal epigenetic changes during differentiation of human skeletal muscle stem cells from obese subjects. *BMC Med* **15**, 39, doi:10.1186/s12916-017-0792-x (2017).

3 Teschendorff, A. E. *et al.* A beta-mixture quantile normalization method for correcting probe design bias in Illumina Infinium 450 k DNA methylation data. *Bioinformatics* **29**, 189-196, doi:10.1093/bioinformatics/bts680 (2013).

4 Johnson, W. E., Li, C. & Rabinovic, A. Adjusting batch effects in microarray expression data using empirical Bayes methods. *Biostatistics* **8**, 118-127, doi:10.1093/biostatistics/kxj037 (2007).

5 Chen, Y. A. *et al.* Discovery of cross-reactive probes and polymorphic CpGs in the Illumina Infinium HumanMethylation450 microarray. *Epigenetics : official journal of the DNA Methylation Society* **8**, 203-209, doi:10.4161/epi.23470 (2013).

6 Price, M. E. *et al.* Additional annotation enhances potential for biologically-relevant analysis of the Illumina Infinium HumanMethylation450 BeadChip array. *Epigenetics Chromatin* **6**, 4, doi:10.1186/1756-8935-6-4 (2013).

7 Du, P., Kibbe, W. A. & Lin, S. M. lumi: a pipeline for processing Illumina microarray. *Bioinformatics* **24**, 1547-1548, doi:10.1093/bioinformatics/btn224 (2008).

8 Gentleman, R. C. *et al.* Bioconductor: open software development for computational biology and bioinformatics. *Genome Biol* **5**, R80, doi:10.1186/gb-2004-5-10-r80 (2004).

9 McCartney, D. L. *et al.* Identification of polymorphic and off-target probe binding sites on the Illumina Infinium MethylationEPIC BeadChip. *Genom Data* **9**, 22-24, doi:10.1016/j.gdata.2016.05.012 (2016).
