# Supplementary material for: VPS39-deficiency observed in type 2 diabetes impairs muscle stem cell differentiation via altered autophagy and epigenetics
Source: Nat Commun. 2021 Apr 23;12:2431. doi: 10.1038/s41467-021-22068-5 (PMC8065135; doi:10.1038/s41467-021-22068-5)
Supplement: Supplementary file 3 — Description of Additional Supplementary Files [file 41467_2021_22068_MOESM3_ESM.pdf]

Description of Additional Supplementary Files

Title: Supplementary Data 1.

Description: mRNA expression levels in human myoblasts and myotubes from controls and individuals with type 2 diabetes (T2D).

Sheets A-B. Comparison of gene expression in A) myoblasts and B) myotubes from controls (normal glucose tolerance (NGT)) versus individuals with type 2 diabetes (T2D). n=13 individuals per group. Statistics were calculated using linear regression analyses adjusted for age, BMI and sex to compare mRNA expression array data between individuals with NGT and T2D and false discovery rate (FDR) below 5% was applied (q<0.05) to correct for multiple testing.

Sheets C-D. Comparison of gene expression in myoblasts versus myotubes from individuals with C) T2D or D) NGT. n=13 individuals per group. Statistics were calculated using paired two-tailed Wilcoxon test and false discovery rate (FDR) below 5% was applied (q<0.05). One gene can be targeted by more than one probe. Thus, the number of unique genes may be lower than the number of unique Illumina IDs in the table. Data are presented as mean±SD.

Title: Supplementary Data 2.

Description: Correlations between gene expression and DNA methylation in human myoblasts. Spearman correlation analysis between gene expression and DNA methylation of CpG sites annotated to the same gene. n=26. Only genes with differential gene expression between controls (normal glucose tolerance (NGT)) and individuals with T2D (see Supplementary Data 1, sheet A) are included.

Title: Supplementary Data 3.

Description: Microarray analysis of gene expression in VPS39-silenced human myoblasts versus controls at day 3 of differentiation.

Sheet A. Genes with significantly different mRNA expression in VPS39-silenced (siVPS39) myoblasts versus negative control (NC). Statistics were calculated using paired two-tailed t-test and false discovery rate (FDR) below 5% was applied (q<0.05). n=6 per group. One gene can be targeted by more than one probe. Thus, the number of unique genes may be less than the number of unique Probe Set IDs shown in the table. Data are presented as mean±SD.

Sheet B. Results of gene set enrichment analysis (GSEA) based on the expression data set in VPS39-silenced myoblasts versus controls and the GSEA preranked module. False discovery rate (FDR) below 5% was applied and gene sets with q<0.05 were considered significant and are presented.

Sheets C-D. Results from PSCAN analysis: Enrichment of transcription factor binding motifs (determined by JASPAR) in the promoter region (1-1000 bp upstream of the transcription start site

(TSS)) of C) downregulated and D) upregulated genes in VPS39-silenced myoblasts versus controls at day 3 of differentiation. PSCAN is a software tool that scans promoter sequences from co-regulated genes, looking for overrepresented motifs describing the binding specificity of known transcription factors (TFs). False discovery rate (FDR) below 5% was applied and motifs with q<0.05 were considered significant and are presented.

Sheets E-F. E) Downregulated and F) upregulated genes in VPS39-silenced myoblasts versus controls on day 3 of differentiation that contains a binding motif for myogenin are shown (See "Myog" in sheet C).

Title: Supplementary Data 4.

Description: Gene expression and DNA methylation in VPS39-silenced myoblasts and controls at day 3 of differentiation. Genes with significant differences in mRNA expression (q<0.05, see Supplementary Data 3, sheet A) and differential DNA methylation with p<0.05 are presented for VPS39-silenced (siVPS39) myoblasts vs. negative control (NC) at day 3 of differentiation. Statistics were calculated using paired two-tailed t-test. n=6 per group. Data are presented as mean ± SD.

Title: Supplementary Data 5.

Description: Gene expression in skeletal muscle from Vps39+/- and wild-type mice.

Sheet A. Genes with different mRNA expression in Vps39+/- vs. wild-type (WT) mice based on p<0.05. Statistics were calculated using unpaired two-tailed t-test. n=12 per genotype. One gene could be targeted by more than one probe and may therefore be presented in the table twice. Thus, the number of unique genes may be lower than the number of unique Probe Set ID. Data are presented as mean±SD.

Sheet B. GO biological process terms assigned to all genes analyzed and genes with differential expression based on p<0.05 (see sheet A). Terms marked with X in the column "Contributing terms" are included in Figure 5e, which describes the frequency of the selected GO terms among the differentially expressed genes (p<0.05) and in Figure 5e. Chi2-tests were used to analyze overrepresentation of differentially expressed genes belonging to a GO term compared with all analyzed genes. One gene could be annotated to multiple GO terms.

Sheet C. Gene set enrichment analysis (GSEA) using the GSEA pre-ranked module on the complete mRNA expression data set in Vps39+/- vs. WT mice 10. This analysis revealed pathways with false discovery rates (FDR) below 5% (q<0.05) in muscle from Vps39+/- vs. WT mice.

Title: Supplementary Data 6.

Description: DNA methylation in human myoblasts versus myotubes from 14 control (NGT) and 14 individuals with type 2 diabetes (T2D).

Sheet A. Average DNA methylation for all analyzed CpG sites in different gene regions and regions in relation to CpG islands. Statistics are based on linear regression analyses and adjusted for age, BMI and sex for individuals with normal glucose tolerance (NGT) vs. T2D and paired two-tailed Wilcoxon test comparing myoblasts and myotubes within the same group. False discovery rate (FDR) below 5% was applied ($q<0.05$).

Sheets B-C. DNA methylation in myoblasts versus myotubes from B) n=14 controls (NGT) and C) n=14 individuals with type 2 diabetes (T2D). Statistics were calculated using paired two-tailed Wilcoxon test and false discovery rate (FDR) below 5% was applied ($q<0.05$). Data are presented as mean±SD.

Sheets D-E. DNA methylation data of CpG sites significant in D) only controls (NGT) and not individuals with type 2 diabetes (T2D) and E) only individuals with T2D and not NGT when comparing the degree of DNA methylation in myoblasts versus myotubes within the same group. Statistics were calculated using paired two-tailed Wilcoxon test and false discovery rate (FDR) below 5% was applied ($q<0.05$). Data are presented as mean±SD.
