# Supplementary material for: VPS39-deficiency observed in type 2 diabetes impairs muscle stem cell differentiation via altered autophagy and epigenetics
Source: Nat Commun. 2021 Apr 23;12:2431. doi: 10.1038/s41467-021-22068-5 (PMC8065135; doi:10.1038/s41467-021-22068-5)
Supplement: Supplementary file 10 — Reporting Summary [file 41467_2021_22068_MOESM10_ESM.pdf]

nature research

Corresponding author(s): Charlotte Ling
Last updated by author(s): Dec 26, 2020

Reporting Summary

Nature Research wishes to improve the reproducibility of the work that we publish. This form provides structure for consistency and transparency in reporting. For further information on Nature Research policies, see our Editorial Policies and the Editorial Policy Checklist.

Statistics

For all statistical analyses, confirm that the following items are present in the figure legend, table legend, main text, or Methods section.

n/a	Confirmed	
☐	☒	The exact sample size (n) for each experimental group/condition, given as a discrete number and unit of measurement
☐	☒	A statement on whether measurements were taken from distinct samples or whether the same sample was measured repeatedly
☐	☒	The statistical test(s) used AND whether they are one- or two-sided<br>*Only common tests should be described solely by name; describe more complex techniques in the Methods section.*
☐	☒	A description of all covariates tested
☐	☒	A description of any assumptions or corrections, such as tests of normality and adjustment for multiple comparisons
☐	☒	A full description of the statistical parameters including central tendency (e.g. means) or other basic estimates (e.g. regression coefficient) AND variation (e.g. standard deviation) or associated estimates of uncertainty (e.g. confidence intervals)
☐	☒	For null hypothesis testing, the test statistic (e.g. F, t, r) with confidence intervals, effect sizes, degrees of freedom and P value noted<br>*Give P values as exact values whenever suitable.*
☒	☐	For Bayesian analysis, information on the choice of priors and Markov chain Monte Carlo settings
☒	☐	For hierarchical and complex designs, identification of the appropriate level for tests and full reporting of outcomes
☐	☒	Estimates of effect sizes (e.g. Cohen's d, Pearson's r), indicating how they were calculated

Our web collection on statistics for biologists contains articles on many of the points above.

Software and code

Policy information about availability of computer code

Data collection	GenomeStudio V2011.1
Data analysis	Expression Console Software v1.4.1.46, PSCAN Web Interface v1.4, R (programming language, v3.4.3), Bioconductor v3.6, lumi package v2.30, BMIQ v1.4, COMBAT (sva package, v3.20), GraphPad Prism v9.0.0

For manuscripts utilizing custom algorithms or software that are central to the research but not yet described in published literature, software must be made available to editors and reviewers. We strongly encourage code deposition in a community repository (e.g. GitHub). See the Nature Research guidelines for submitting code & software for further information.

Data

Policy information about availability of data

All manuscripts must include a data availability statement. This statement should provide the following information, where applicable:

- Accession codes, unique identifiers, or web links for publicly available datasets
- A list of figures that have associated raw data
- A description of any restrictions on data availability

DATA AVAILABILITY
mRNA expression and DNA methylation data that support the findings of this study have been deposited in the NCBI Gene Expression Omnibus (GEO) and the LUDC repository (https://www.ludc.lu.se/resources/repository). mRNA expression data from VPS39-silenced (siVPS39) and control human myoblasts are available in GEO with accession number GSE157345 [https://www.ncbi.nlm.nih.gov/geo/query/acc.cgi?acc=GSE157345]. mRNA expression data from skeletal muscle from Vps39+/- and control mice are available in GEO with accession number GSE157342 [https://www.ncbi.nlm.nih.gov/geo/query/acc.cgi?acc=GSE157342]. mRNA expression data from myoblasts (accession number LUDC2020.08.9) and myotubes (accession number LUDC2020.08.10), and DNA methylation data from myoblasts (accession

number LUDC2020.08.11) and myotubes (accession number LUDC2020.08.12) from individuals with T2D and control individuals, as well as DNA methylation data from siVPS39 and control human myoblasts (accession number LUDC2020.08.13) are deposited in the LUDC repository (https://www.ludc.lu.se/resources/repository) and are available upon on request. Source data underlying relevant panels in Fig. 1-6, and Supplementary Fig. 1-2 and 4-6 are provided with this publication.

The following figures have associated raw data based on genome-wide DNA methylation and expression data:
Figure 1c-e, Figure 2b-g , Figure 4f-g, Figure 5e-f, Figure 7b-e, Supplementary Figure 1a-c, Supplementary Figure 4g, Supplementary Figure 5c-g

Field-specific reporting

Please select the one below that is the best fit for your research. If you are not sure, read the appropriate sections before making your selection.

- [x] Life sciences
- [ ] Behavioural & social sciences
- [ ] Ecological, evolutionary & environmental sciences

For a reference copy of the document with all sections, see nature.com/documents/nr-reporting-summary-flat.pdf

Life sciences study design

All studies must disclose on these points even when the disclosure is negative.

Sample size	Sample size was determined based on power calculations. These were made based on our previous data, where we have analyzed DNA methylation and gene expression in human muscle samples/cells.
Data exclusions	One sample (T2D Day 0) was excluded from Western blot analysis of DNMT3B in T2D and NGT muscle cells due to poor technical quality. Likewise, one sample (WT female) was excluded from Western blot analysis of ATG5 in Vps39+/- and WT mouse skeletal muscle due to poor technical quality.
Replication	We have used several different methods/models to test the reproducibility of our data. For example, we use both human samples from people with type 2 diabetes, who show reduced VPS39 expression, and we then silenced VPS39 in both human muscle cells and in an animal model to assess if we see similar/reproducible results in "all three models with reduced VPS39". There are several occasions in the paper where we test if data can be reproduced by different methods and different models. For example key autophagy markers (LC3B, p62, and LAMP1 and LAMP2) were studied in VPS39 silenced and control cells using both automated high-content screening (HCS) and Western blot analyses . The majority of attempts at replication were successful, except discrepancies in LAMP1/2 levels between the HCS and Western blot assays that may depend on that these two separate methods detect different aspects of the protein dynamics, and that HCS is able to detect more subtle changes in LAMP1/2 levels by measuring spot number and spot area per cell compared to Western blot. This is discussed in the manuscript. We state the number of independent samples in each legend and all experiments were performed using at least three independent biological replicates.
Randomization	Samples were randomized to chips for expression and methylation arrays.
Blinding	During the genome-wide DNA methylation and gene expression experiments the samples were anonymous to the technicians who run the experiments and hence they did not know which group samples belonged to. However, during for example qPCR and Western blot analysis, the technician needed to know the order the samples were loaded on plates and gels and then blinding was not possible.

Reporting for specific materials, systems and methods

We require information from authors about some types of materials, experimental systems and methods used in many studies. Here, indicate whether each material, system or method listed is relevant to your study. If you are not sure if a list item applies to your research, read the appropriate section before selecting a response.

Materials & experimental systems

n/a	Involved in the study	
☐	☒	Antibodies
☐	☒	Eukaryotic cell lines
☒	☐	Palaeontology and archaeology
☐	☒	Animals and other organisms
☐	☒	Human research participants
☒	☐	Clinical data
☒	☐	Dual use research of concern

Methods

n/a	Involved in the study	
☒	☐	ChIP-seq
☒	☐	Flow cytometry
☒	☐	MRI-based neuroimaging

Antibodies

Antibodies used	The following primary antibodies were used for western blotting: mouse anti-DNMT3B (1:200, Santa Cruz sc-376043), mouse anti-EZH2 (1:1000, Cell Signaling #3147), mouse anti-LAMP1 (0.3 ug/mL, DSHB H4A3), mouse anti-LAMP2 (0.3 ug/mL, DSHB H4B4), mouse anti-MYOD (1:200, Santa Cruz sc-377460), mouse anti-myosin (1:1000, Sigma AldrichM4276), mouse anti-VPS39

(1:200, Santa Cruz sc-514762), rabbit anti-Akt (1:1000, Cell Signaling #9272), rabbit anti-phospho-Akt Thr308 (1:1000, Cell Signaling #9275), rabbit anti-phospho-Akt Ser473 (1:1000, Cell Signaling #9271), rabbit anti-ATG5 (1:1000, Cell Signaling #12994), rabbit anti-DNMT1 (1:1000, Novus Biologicals NB100-264), rabbit anti-DNMT3A (1:250, Sigma Aldrich HPA026588), rabbit anti-DNMT3B (1:250, Sigma Aldrich HPA001595), rabbit anti-FBN2 (0.4 ug/mL, Novus Biologicals NBP1-88169), rabbit anti-GSK3a/b (1:1000, Thermo Fisher Scientific 44-610), rabbit anti-phospho-GSK3a/b Ser21/Ser9 (1:1000, Cell Signaling #9331), rabbit anti-H3 (1:4000, Abcam ab1791), rabbit anti-acetylated-H3 (1:500, Millipore 06-599), rabbit anti-HAT1 (1:200, sc-366092), rabbit anti-HDAC4 (1:1000, Abcam ab12172), rabbit anti-HDAC5 (1:1000, Cell Signaling #20458), rabbit anti-LC3B (1:1000, Novus Biologicals NB100-2220), rabbit anti-MEF2C (1:1000, Abcam ab211493), rabbit anti-p300 (1:200, Santa Cruz sc-584), rabbit anti-p62 (1:1000, Abcam ab91526), rabbit anti-TBC1D4 (1:1000, Millipore 07-741), rabbit anti-phospho-TBC1D4 Thr642 (1:1000, Thermo Fisher Scientific 44-1071G), sheep anti-MAEA (1 ug/mL, R&D Systems AF7288). Secondary antibodies were raised in goat and conjugated to HRP: anti-mouse (1:5000, Bio-Rad 170-6516), anti-rabbit (1:10000, Cell Signaling #7074), anti-sheep (1:2500, Dako P0163).
The following primary antibodies were used for immunohistochemistry: mouse anti-LAMP1 (3 ug/mL, DSHB H4A3), mouse anti-LAMP2 (3 ug/mL, DSHB H4B4), mouse anti-myosin (1:50, DSHB MF 20), rabbit anti-LC3B (1:1000, Novus Biologicals NB100-2220), rabbit anti-p62 (1:500, Abcam ab91526). Secondary antibodies were raised in goat and conjugated to Cy5 (anti-mouse, 1:800, Jackson ImmunoResearch 115-225-146) or Cy3 (anti-rabbit, 1:800, Jackson ImmunoResearch 111-165-144).

Validation

All antibodies are commercially available and have been characterized by the manufacturers (and in several publications) for their reactivity in the appropriate species and for their compatibility to be used with the respective application. Conditions for blocking and antibody dilutions are stated in Supplementary Table 3. siRNA-silenced samples were used for validation of anti-VPS39 (sc-514762, Figure 1h), anti-MAEA (AF7288, Supplementary Figure 1e), anti-FBN2 (NBP1-88169, Supplementary Figure 1f) and anti-DNMT3B (HPA001595, Supplementary Figure 5a) antibodies. Additionally, antibodies were characterized by the expected regulation pattern in positive/negative control samples. For Western blot we used molecular weight markers to identify the band(s) that migrated at the expected size of each respective protein analyzed. Validation details are also available on the manufacturers' websites.

Eukaryotic cell lines

Policy information about cell lines

Cell line source(s)	C2C12 (ATCC® CRL1772™) myoblasts
Authentication	It was verified that these cells are myoblasts with the ability to differentiate into myotubes by morphological visualization under the light microscope. The company (ATCC) also included a Certificate of Analysis (COA) for batch-specific test results. However, this cell line was not authenticated using any additional authentication techniques.
Mycoplasma contamination	Cells were tested negative for mycoplasma.
Commonly misidentified lines (See ICLAC register)	No commonly misidentified cell lines were used in the study.

Animals and other organisms

Policy information about studies involving animals; ARRIVE guidelines recommended for reporting animal research

Laboratory animals	We used wild-type (WT) and Vps39+/- mice, on a C57BL/6J background. Both male and female 4-month old mice were included in this study. Animals were kept in the following controlled housing conditions; 21–22 °C, 55–65% humidity and 12-h light/12-h dark cycle.
Wild animals	No wild animals were used in the study.
Field-collected samples	No field collected samples were used in the study.
Ethics oversight	All animal experiments were performed with permission of the Animal Ethics Committee of the University of Gothenburg, in accordance with the legal requirements of the European Community (Decree 86/609/EEC).

Note that full information on the approval of the study protocol must also be provided in the manuscript.

Human research participants

Policy information about studies involving human research participants

Population characteristics	Individuals with Type 2 Diabetes (n=14) were selected to obtain a group with a similar gender composition, age, and BMI as in the control group (n=14). The control group included, 7 males and 7 females, they were 54.2 +/- 6.8 years old and their BMI was 24.7 +/- 2.4 kg/m2. The individuals with type 2 diabetes included, 7 males and 7 females, they were 58.1 +/- 6.6 years old and their BMI was 26.6 +/- 3.1 kg/m2. More clinical characteristics of these human donors of muscle cells are described in table 1.
Recruitment	Participants were primarily recruited from advertisements in local newspapers and three of the participants with impaired glucose tolerance (type 2 diabetes) were recruited from a register of known test subjects at Steno Diabetes Centre. It is possible that individuals with type 2 diabetes who are interested in research chose to participate in this study and that may contribute to a self-selection bias or other biases that may impact our results. However, it is impossible to dissect how such bias would impact our results.

Ethics oversight	The study was approved by the local ethics committee (National Committee on Health Research Ethics (DNVK) KF 01-141/04) and followed the principles of the Helsinki declaration. All study participants had provided informed written consent before any experiments.

Note that full information on the approval of the study protocol must also be provided in the manuscript.
